# Real-time processing of high-throughput quantitative phase microscopy data using a Jetson Orin Nano

**DOI:** 10.1117/1.BIOS.3.1.012902

**Published:** 2025-10-21

**Authors:** Wan Wang, Robert E. Highland, Justin Fan, David A. Miller, Adam Wax

**Affiliations:** Duke University, Department of Biomedical Engineering, Durham, North Carolina, United States

**Keywords:** quantitative phase microscopy, holographic cytometry, high-throughput, real-time processing, digital refocusing

## Abstract

**Significance:**

Quantitative phase microscopy (QPM) is a holographic imaging technique often applied to studying cell morphology. To advance QPM for clinical applications, high-throughput implementations have been developed to allow imaging of thousands of cells at a time.

**Aim:**

To meet the needs of processing raw data and creating QPM holographic images, higher throughput processing methods are needed. Here, we report on the use of a system-on-module approach for QPM data processing.

**Approach:**

We have developed a real-time processing pipeline that leverages the parallel processing capabilities of the NVIDIA Jetson Orin Nano to implement processing of cell data. We demonstrate this pipeline on a holographic cytometry (HC) system, a high-throughput QPM implementation. The CUDA processing algorithm enables the generation of QPM data from raw interferograms followed by phase unwrapping, cell segmentation, and refocusing.

**Result:**

We captured, processed, and analyzed 107,631 red blood cell images. The processing speed reaches 1200 cells/s in the speed test. Benchmarking shows that real-time refocusing maintains a high degree of structural similarity to the traditional refocusing method.

**Conclusion:**

The result demonstrates that our pipeline could accelerate the statistical analysis of cell populations. We expect this study to benefit the development of a portable, low-cost HC system.

Statement of Discovery This work achieves real-time processing of quantitative phase microscopy on an embedded GPU platform at a speed of up to 1200 red blood cells per second. The result demonstrates the capability for high-throughput statistical analysis of cell populations.

## 1 Introduction

Flow cytometry (FC) is a label-free, high-throughput tool for rapid assessment and quantification of large cell populations but lacks imaging capabilities and key measurement parameters for accurate classification of a large number of cell types.^1^ Imaging flow cytometry (IFC) has since been introduced and widely adopted, increasing the technique’s diagnostic capabilities.^2^ However, there are limitations associated with fluorescent IFC techniques, such as the influence of fluorescent antigens on cell viability and quantification limitations. Therefore, recent advances have been made to develop label-free IFC, utilizing techniques such as Raman scattering,^3^ brightfield,^2^ and interferometric microscopy.^4^ Quantitative phase microscopy (QPM) is an interferometric imaging method that measures the phase delay of light that passes through a thin, weakly scattering sample. Intracellular structures cause refractive index variations across the cell, which can be measured by phase delay and reconstructed into phase images using QPM. Our group previously introduced a holographic IFC technique, which implements QPM as holographic cytometry (HC), to produce high-throughput cell imaging data using microfluidic elements and stroboscopic illumination in an off-axis Mach–Zehnder interferometer.^5^ Over the past few years, we have used HC for the analysis and classification of breast cancer cell lines and red blood cells (RBCs).^6^^,^^7^

Although QPM continues to advance as a method to noninvasively study intracellular structural dynamics, high-speed phase processing becomes highly advantageous for increasing its throughput. In many QPM setups, phase extraction usually involves either a 2D Fourier transform or a Hilbert transform. In addition, off-axis QPM techniques require a mathematical phase unwrapping algorithm to remove 2π phase ambiguity across the image.^8^ These two steps can be computationally intensive, typically involving lengthy post-processing. Furthermore, these steps can become quite laborious when dealing with a large dataset such as those acquired with HC, where over 100,000 cell images are routinely obtained for each sample.^5^ Although image acquisition is quick in HC, a PC/MATLAB interface takes several hours to extract phase information and perform unwrapping on hundreds of thousands of raw interference images (96×4096  pixels each).

A solution to this processing bottleneck can be found using the CUDA programming language and parallel computing devices to compartmentalize computationally intensive processing steps across multiple graphics processing unit (GPU) cores. This approach significantly accelerates the processing times that are typically handled across CPU cores. Pham et al.^9^ previously implemented GPU processing for QPM, which achieved real-time quantitative phase imaging by advancing quantitative phase processing rates beyond video rate (30 fps). Alternatively, Dardikman et al.^10^ utilized a GPU/CUDA infrastructure to accelerate tomographic phase processing. However, these processing approaches are designed for imaging of stationary samples or lower throughput versions of holographic cytometry. We now seek to expand this type of processing architecture to match the data acquisition speeds of a high-throughput holographic cytometry system.

In this paper, we report on the development of a high-speed, CUDA-based QPM processing algorithm to further the diagnostic capability of our HC system. We utilized the parallel computing of an NVIDIA Jetson Orin Nano, an embedded GPU platform, to realize a real-time phase processing pipeline. In addition to processing the raw interferogram to create QPM images, the algorithm also implements high-speed digital refocusing and morphological parameter analysis to analyze RBCs at high throughput. The system processing algorithms, performance, and RBC analysis are described here for the HC system.

## 2 Method

### 2.1 System Setup

**Fig. 1:**
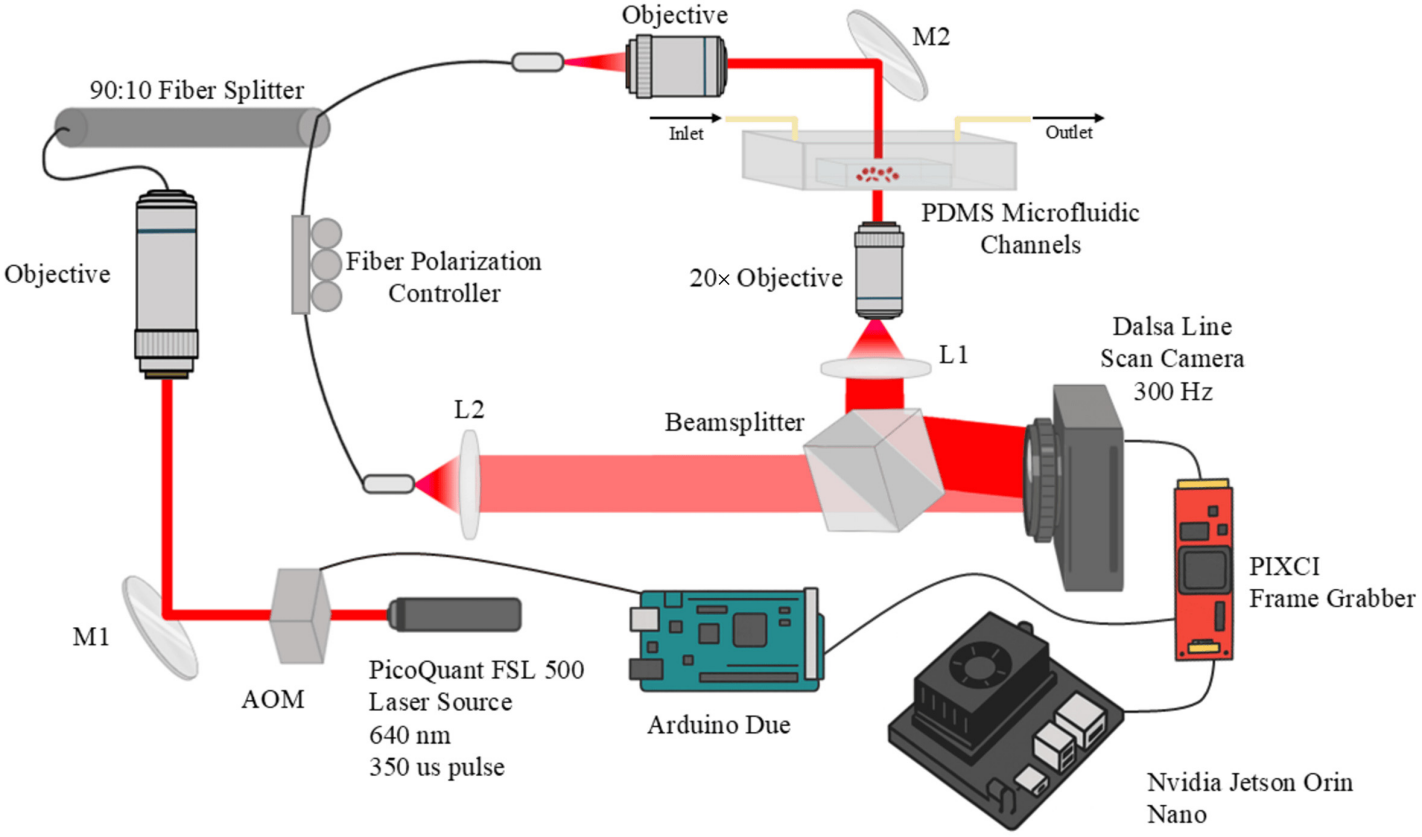
Holographic cytometry setup. Light from the pulsed laser source is divided into sample and reference arms using a fiber coupler. The sample arm light is passed through the microfluidic element containing the sample and imaged onto a line scan camera using a 20× objective. The reference arm light is delivered to the sensor with a different angle of propagation to implement off-axis interferometry.

The experimental setup is based on the HC system described previously (Fig. 1).^5^^,^^7^ Briefly, the system uses a 20× sample objective, a 640 nm pulsed diode laser (PicoQuant FSL 500, Berlin, Germany), and a high-speed line-scan camera (Dalsa HS-40-04K40-00-R, Waterloo, California, United States), implemented in an off-axis Mach–Zehnder interferometer. The numerical aperture (NA) is 0.4, corresponding to the lateral resolution of 0.976  μm. The pixel size of the acquired image is 0.203  μm. Thus, the spot diameter on the detector is ∼5  pixels. The camera acquires images at 300 frames per second (fps) and is synchronized with an acousto-optic modulator, which modulates the laser at a 350  μs pulse duration. This synchronization is performed to allow stroboscopic illumination, eliminating the motion blurring of flowing cells. The RBC sample was flowed through a previously described custom microfluidic channel,^5^^,^^7^ using a programmable syringe pump (NE-4000, New Era Pump Systems, Farmingdale, New York, United States).

We used an NVIDIA Jetson Orin Nano Developer Kit (NVIDIA, Santa Clara, California, United States) to both control the system and capture the data. The specifications are shown in Table 1. A frame grabber (PIXCI® mf2280; Epix, Buffalo Grove, Illinois, United States) installed in the PCIe ×4 port of the developer kit was used to capture raw data from the camera. The exposure trigger was routed to an Arduino Due (Arduino, Monza, Italy), which translated the trigger signal for the AOM.

**Table 1:** Specifications of Jetson Orin Nano Developer Kit.

Features	
Model	Jetson Orin Nano 8 GB (Jetpack 6.2)
CPU	6-core Arm^®^ Cortex^®^-A78AE v8.2 64-bit CPU
CPU max frequency	1.7 GHz
GPU	1024-core NVIDIA Ampere architecture GPU with 32 Tensor Cores
GPU max frequency	1020 MHz
Power consumption	25 W

### 2.2 Real-Time QPM Processing Pipeline

**Fig. 2:**
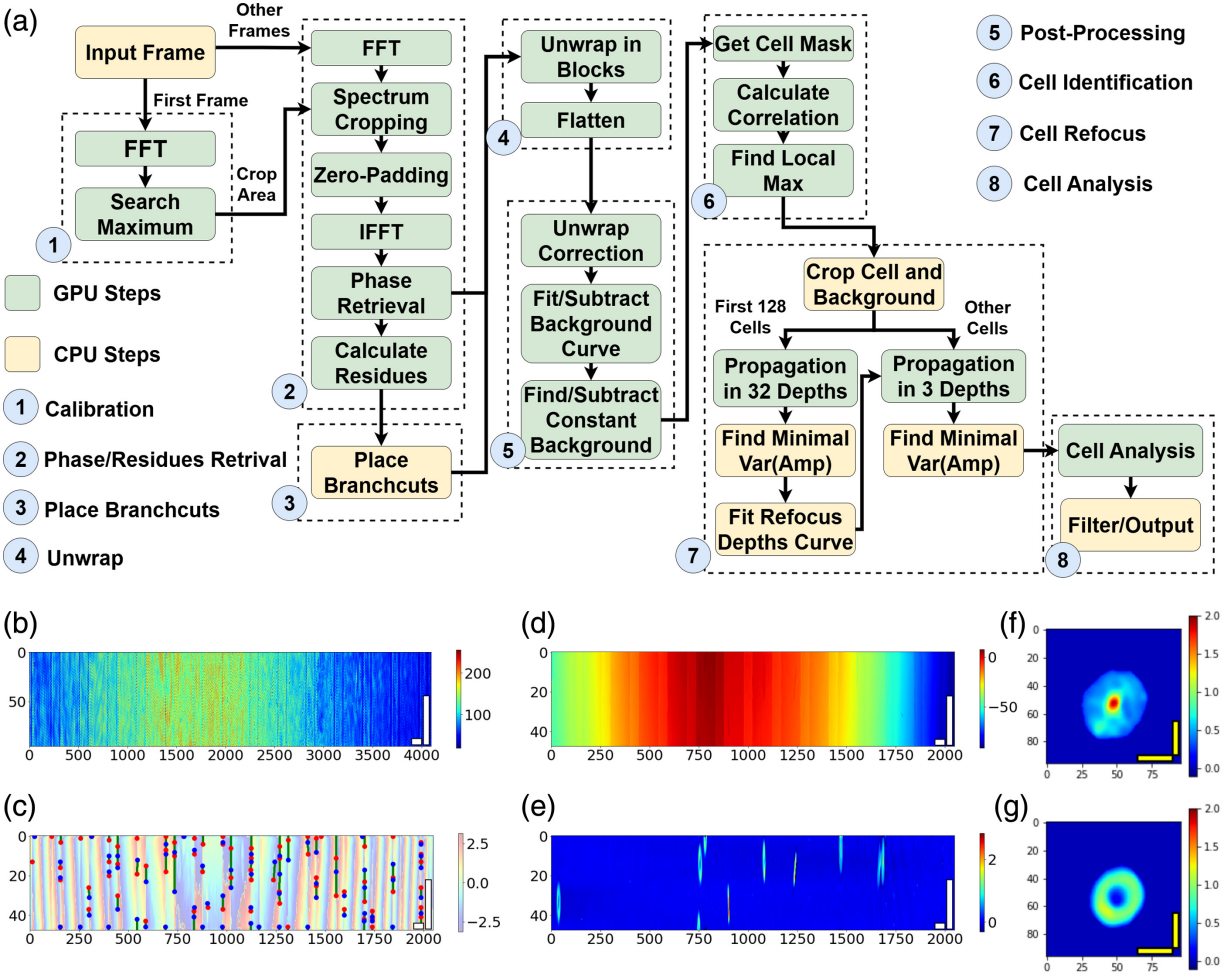
(a) Flowchart of the HC processing pipeline, where each dashed box indicates a processing thread; (b) typical input interferogram (96×4096  pixel); (c) wrapped phase map together with residues and branch cuts; (d) unwrapped phase map; (e) background subtracted phase map; (f) phase map of RBC before refocusing; (g) phase map of RBC after refocusing [white scale bars: 20  μm in x-axis, 10  μm in y-axis; yellow scale bars: 5  μm; colorbar of panel (b): intensity (a.u.); colorbar of panels (c)–(g): phase (rad)].

We designed a real-time processing pipeline to extract phase images and basic morphological parameters of individual cells. Figure 2(a) describes the flowchart of this processing pipeline, where each dashed box depicts a task thread. The threads are run in parallel to increase the system throughput. Because the Jetson Orin Nano utilizes a unified memory shared by both the CPU and GPU, we can execute different tasks on the CPU and GPU with negligible delays in data transmission. Figures 2(b)–2(g) show some important intermediate outputs of the pipeline.

#### 2.2.1 Spectrum calibration and phase retrieval

In off-axis QPM, the cross-correlation term is positioned in the high-frequency domain and can be extracted by Fourier transforming the interferogram image, cropping the region of interest (ROI), and then conducting an inverse FFT. As the beam angle may change in different experiments and shift the desired ROI, we identify the cropping region by searching for the local maximum of magnitude in the 2D Fourier transform of the first captured frame. After calibration, an FFT is applied to each frame, and an 874×20  pixel ROI is cropped from the spectrum. The cropped ROI is then placed at the center of the Fourier domain and inverse transformed. Two different zero-paddings are applied to obtain 2048×48  pixel (half resolution) and 4096×96  pixel (full resolution) reconstructed complex optical field. The phase information from the half-resolution complex data is extracted and used for the following steps. The full-resolution data are retained in memory for cell segmentation and refocusing.

#### 2.2.2 Phase unwrapping

The wrapped phase recovered from the interference fringes can be expressed as

ψ(x,y)=Wrap(φ(x,y))=φ(x,y)+2πk(x,y), (1)

where φ(x,y) is the unwrapped phase, ψ(x,y)∈(−π,π] is the obtained wrapped phase, and k(x,y) is an integer of wrapping time. Goldstein’s algorithm is a classic phase unwrapping method that balances stability and execution efficiency,^11^ and the capability to implement in parallel on the GPU has been proven by Pham et al.^9^ In this study, a parallel Goldstein’s algorithm was designed to conduct a fast phase unwrapping on the Jetson Orin Nano.

The residues are identified by calculating the pixel-wise integration over each 4-pixel loop

$$R(x,y)=\text{Sign}(\begin{array}{c} \Delta (x,y,x+1,y)+\Delta (x+1,y,x+1,y+1)\\ +\Delta (x,y+1,x+1,y+1)+\Delta (x,+1,x,y) \end{array}),$$ (2)

where

$$\Delta (x_{1},y_{1},x_{2},y_{2})=\text{Wrap}(\psi (x_{2},y_{2})-\psi (x_{1},y_{1})).$$ (3)

In the processing pipeline, this step was conducted on the GPU with the half-resolution phase map from the previous step. The phase retrieval and residue identification are applied in the same thread to simplify the process.

Branch cuts are the shortest connections of positive and negative residues (or residues to image edges) that unwrapping pathways cannot cross. As the placed branch cuts will change the properties of the connected residues and affect the following placements, parallelization of this task is a challenge. As Jetson’s CPU and GPU share the same memory, and thus, data transfer latency is very low, we directly run the branch cut placement of each residue map on a single CPU thread. For further acceleration, we run two branch cut placement threads simultaneously, each processing a separate frame. The number of threads can be further increased for more complex wrapped phase maps.

**Fig 3:**
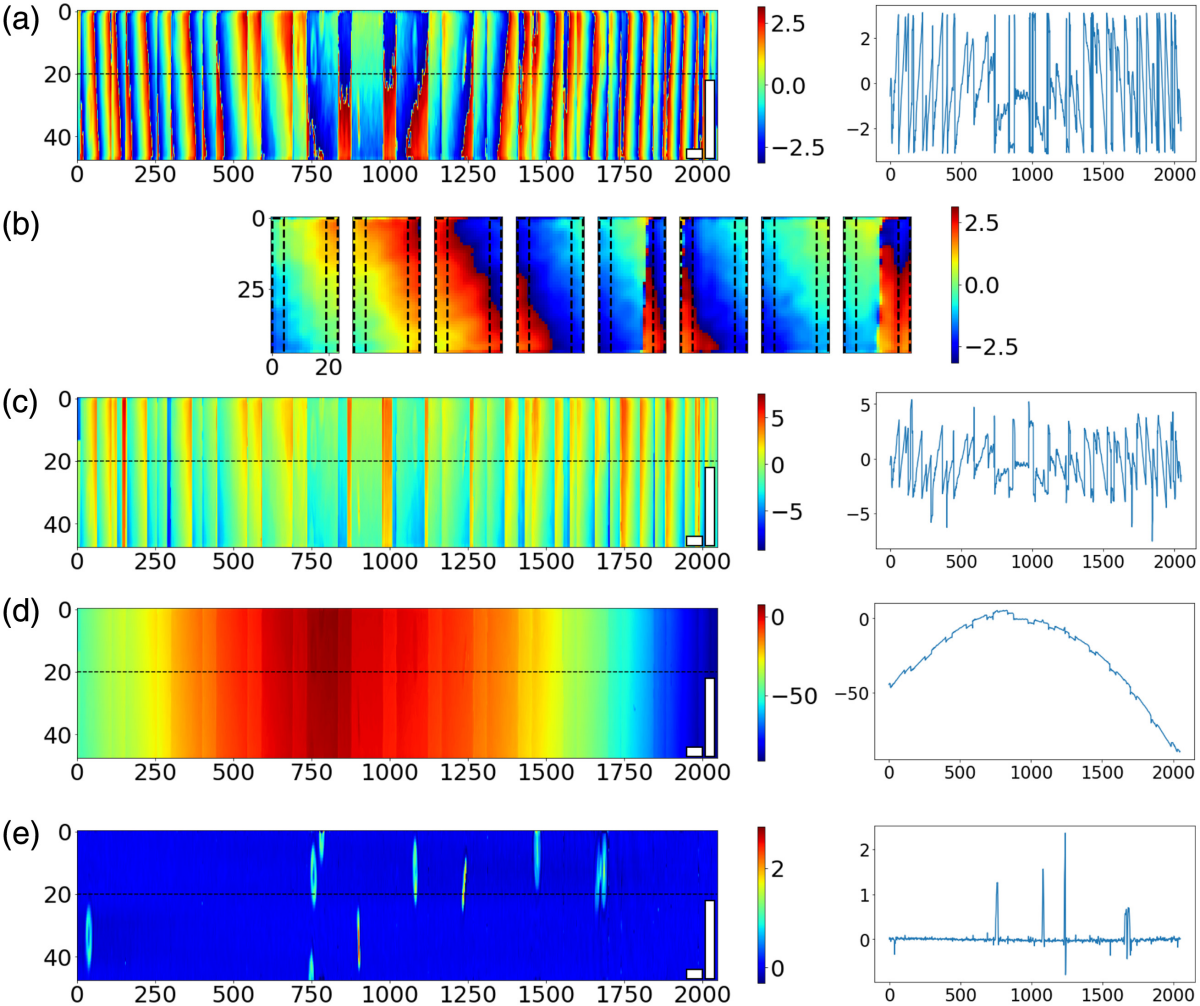
(a) Wrapped phase map; (b) wrapped phase map in blocks, where the dashed box indicates the overlapping regions; (c) unwrapped phase map before flattening; (d) unwrapped phase map after flattening; (e) background subtracted phase map [white scale bars: 20  μm in x-axis, 10  μm in y-axis; colorbars: phase (rad)].

Goldstein’s method uses the flood fill algorithm to perform phase unwrapping. It starts with an initial pixel and unwraps each pixel based on another unwrapped pixel adjacent to it, which can be expressed by

$$\varphi (x,y)=\varphi (x^{\prime },y^{\prime })+\Delta (x^{\prime },y^{\prime },x,y),$$ (4)

where $\varphi (x^{\prime },y^{\prime })$ is the phase value of an adjacent unwrapped pixel. Pixels on branch cuts will not be used as the reference to unwrap the nearby pixels. As the pixels need to be unwrapped sequentially along the path, parallelization of this task is challenging. In the pipeline, each 2048×48  pixel frame [Fig. 3(a)] is split into 128 20×48  pixel blocks along the x-axis [Fig. 3(b)], with a 4×48  pixel overlap between every two adjacent blocks. Then, phase unwrapping is performed in parallel, where each GPU thread unwrapped a block. After all blocks are unwrapped, the mode of the phase difference is calculated in the overlapping area to determine the phase difference between adjacent blocks and flatten the phase when merging the blocks. Figures 3(c) and 3(d) show the phase maps before and after flattening. One particular concern for the algorithm implemented here is the presence of step phase wraps at the edges of the microfluidic channels. As the channel walls resulted in a phase delay larger than π, unwrapping errors will occur at the channel boundaries. These errors are removed in the following post-processing steps. To further improve parallelization and increase throughput, 20 frames are unwrapped simultaneously.

#### 2.2.3 Post-processing

In the post-processing thread, the first step is to correct the phase unwrapping error by searching for and correcting any phase value with a deviation greater than 2π along the time-axis of 40 frames. The unwrapped phase image contains two types of background signals. The first one is introduced by the variation in optical paths across the sample beam, which can be fit by a 2D third-order polynomial surface.^5^ Due to the aspect ratio of the image, the length of the ROI is much longer than its width, so it is effective to just use a third-order polynomial curve fitting for each row instead of the whole 2D plane, which simplifies computation. Due to optical power variation and ambient vibrations, the background phase has high-frequency fluctuations, which can also be suppressed by subtracting the fitted plane. The second background is introduced by the phase delay from the channel walls and artifacts due to dust on the chip. These constant phase delays can be determined by calculating the pixel-wise phase difference along the time-axis. Any remaining unwrapping error will also be identified as background and subtracted. The pipeline conducted background searching and subtraction among 40 frames as a batch. Figure 3(e) shows a post-processed frame, where the background was correctly removed, and only RBCs were observable. Note that the aspect ratio of the ROI makes the RBCs appear elongated in this view.

#### 2.2.4 Cell identification

**Fig 4:**
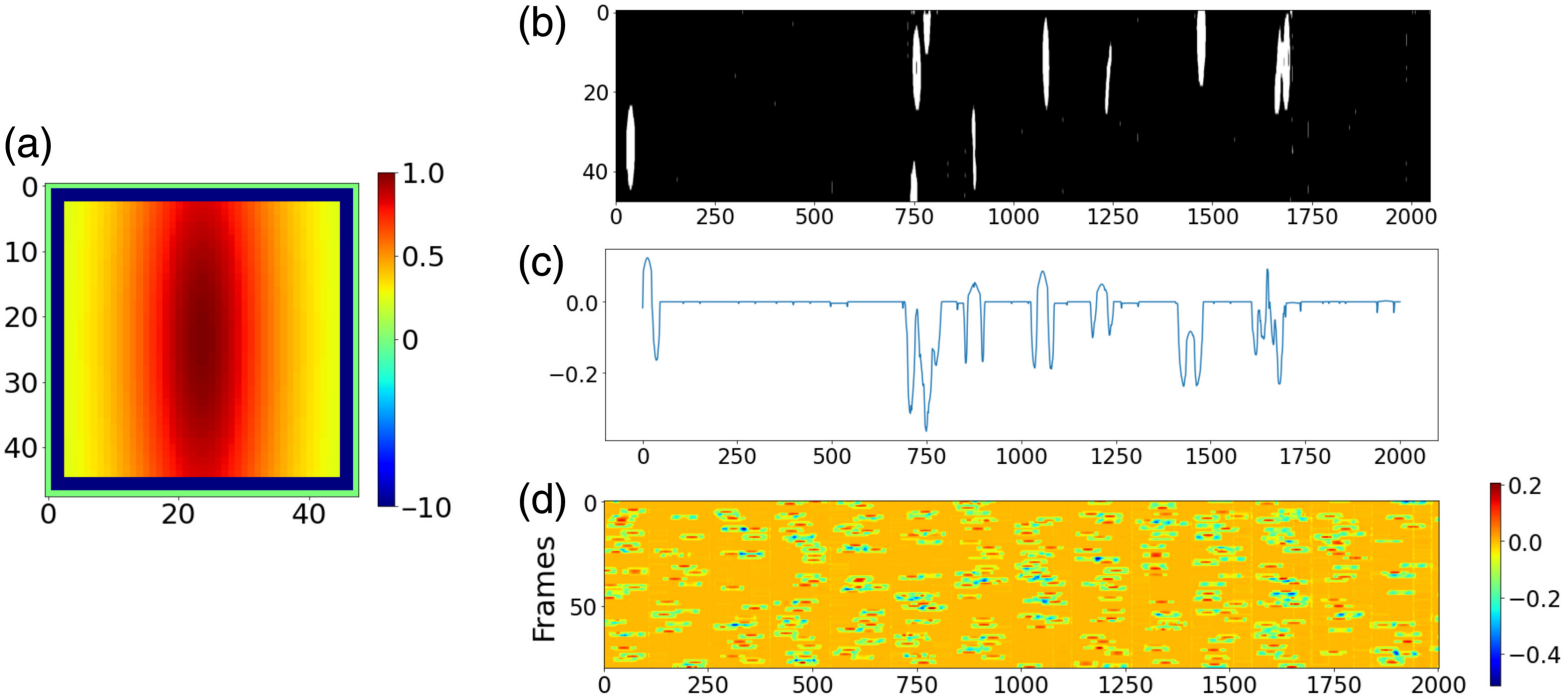
(a) Cell identification kernel; (b) binary mask of processed phase map; (c) correlation of the kernel and mask along x-axis; (d) correlation map of 80 frames. [colorbars of panel (a): value of kernel (a.u.); colorbars of panel (d): correlation (a.u.)].

We developed a cell identification kernel to efficiently segment flowing RBCs [Fig. 4(a)]. A binary mask is generated by applying a threshold (0.3 rad) to the post-processed phase maps, as shown in Fig. 4(b), and its correlation to the kernel is calculated along the x-axis. Figure 4(c) plots the correlation for a given mask. The kernel’s edge is set to a significant negative value to reject cells at the edge of the field of view (FOV). The identification thread calculates 80 frames as a batch and generates an 80-point correlation map [Fig. 4(d)], on which each detected object produces a speckle. By searching for the local max of the speckles, the position where the object occurred at the center of the FOV can be determined. This method can be accelerated by a GPU via FFT. Furthermore, with this approach, motion tracking to avoid capturing repeated objects is unnecessary. After finding the local max on the GPU, the correlation map is sent to the CPU to queue extracted cells in sequence. The time-axis of the correlation map is also searched to find the zero-correlation position and determine the empty background for cell refocusing.

#### 2.2.5 Digital cell refocusing

As it is difficult to manually focus every cell at high speed and wide FOV, digital refocusing is applied on each cell based on the full-resolution complex image from the phase retrieval step. Both 96×96  pixel regions containing the cell and background are cropped, and an angular spectrum propagation is applied to each field.^12^ As the interval between the background and cell frames was only tens of milliseconds, the perturbation of the background is negligible, and the background can be removed by performing complex number division. Pham et al.^13^ proved that the phase delay of less than 2π from thin objects can be extracted by directly subtracting the phase of the object and the background. Therefore, phase unwrapping is not required at this step. In every experiment, the refocus distance is calibrated using the first 128 cells. Past research has shown that the best refocus distance can be determined by minimizing the variance of the amplitude term.^14^^,^^15^ Therefore, the propagation algorithm is applied at 32 different distances from −24 to 24  μm, and the distance of minimal amplitude variance is recorded, together with the x-axis position of the cell. Based on the record of the first 128 cells, a second-order polynomial curve of x-position and refocus distance is fitted. For each following cells, refocusing is performed at three different depths. The first depth that matched the value is given by the calibrated curve based on the cell’s x-position, and the other depths are positioned above and below by one standard deviation of the regression. The refocused cell with minimized amplitude among the three is selected as the result. Compared with traditional refocusing methods on individual cells, this pre-calibrated method reduces the computation time for optical field propagation, enabling real-time refocusing.

#### 2.2.6 Cell analysis

The refocused cell images are post-processed by subtracting the offset of the background, where the offset is determined by averaging the 8×8  pixel region of each corner. Then, a threshold is applied to obtain the binary mask of the cell. Morphological erosion and dilation are performed to filter out any pixels with small degrees of noise. The background region is removed using the binary mask, and then, four basic morphological parameters are calculated for each cell: (1) projection area, (2) optical volume (OV), (3) eccentricity of the fitted ellipse, and (4) circularity. The projection area is the sum of the binary mask multiplied by the pixel area. OV^12^ is expressed by the sum of the optical path differences over the masked area, where the optical path difference can be obtained from the phase map and wavelength. OV can be expressed as

$$\mathrm{OPL}=\Delta n(x,y)*h(x,y)=\varphi (x,y)*\frac{\lambda }{2\pi },$$ (5)

$$\mathrm{OV}=dA*\sum \mathrm{OPL}=dA*\sum \varphi (x,y)*\frac{\lambda }{2\pi }.$$ (6)

The major and minor axes of the ellipse are obtained through the image moments of the mask and are used to calculate the eccentricity and ellipse area. The circularity is calculated by the given formula:

$$e=4\pi *\frac{C^{2}}{A},$$ (7)

where C and A are the perimeter and area, respectively. Thresholds are used to filter nonRBC objects based on these parameters. The selected RBC images are saved in the TIFF format on the SSD of the Jetson, and their morphological parameters are recorded in a CSV file.

### 2.3 RBC Data Acquisition

Fresh whole RBC samples from healthy subjects were provided by Duke Hematology and centrifuged at 6000 rpm for 5 min. After removing the plasma layer, 50  μL of the packed RBC sample was suspended in 5 mL of 20% bovine serum albumin (BSA). The BSA served as a healthy storage medium for the cells and provided a sufficient refractive index difference between medium and sample for QPM. The diluted blood sample was injected into the microfluid chip at a rate of 30  μL/min. For each sample, five datasets were captured and processed, each with 10,000 frames, which contained the raw interferograms, processed phase videos, and cell images. Calibration was applied at the beginning of each data collection procedure as described above.

### 2.4 Execution Speed Test

To better evaluate the processing speed of the pipeline and estimate the maximum throughput, a speed test was performed using the raw QPM data with different RBC concentrations. Considering that it is difficult to precisely control the cell concentration in experiments, different cell concentrations were simulated by ignoring or repeatedly processing a certain proportion of cells in the same dataset. For each dataset, 10,000 frames were processed, and the average execution time of each step was recorded. A pure QPM processing experiment was also performed, which neglected the cell identification and analysis steps to compare our QPM reconstruction speed with other studies.

### 2.5 RBC Data Evaluation

To evaluate the quality of the real-time processed RBC data, a refocusing of the same cells in the dataset using traditional methods was performed by minimizing the amplitude variance of each cell with an accuracy of 0.1  μm.^15^ This refocusing information was then used to determine the accuracy of our real-time refocusing results. For these precisely refocused cell images, five morphological parameters were extracted and compared with the morphological parameters obtained for the RBCs without real-time refocusing. The morphological parameters used were: (1) OV, (2) projection area, (3) mean OPL, (4) eccentricity, and (5) circularity. The root mean square error (RMSE) of the OPL map and the structural similarity index measure (SSIM) of each cell image were also compared. SSIM is given by the following equation^16^:

$$\mathrm{SSIM}(x,y)=\frac{\left(2\mu_{x}\mu_{y}+c_{1})(2\sigma_{xy}+c_{2}\right)}{\left(\mu_{x}^{2}+\mu_{y}^{2}+c_{1})(\sigma_{x}^{2}+\sigma_{y}^{2}+c_{2}\right)},$$ (8)

where $\mu_{x}$ and $\mu_{y}$ are the means of input images, $\sigma_{x}^{2}$ and $\sigma_{y}^{2}$ are the variances of input images, $\sigma_{xy}$ is the covariance, $c_{1}=2.22E-3$ and $c_{2}=2.00E-2$ are constants chosen to stabilize the division by a weak denominator. The RBC images were compared before and after refocusing on the same dataset and performing the same validation.

## 3 Results

### 3.1 Imaging of RBCs

In imaging the RBC sample, the system identified 115,695 objects from the 50,000 frames captured at 300 fps, which provided an acquisition speed of 694.17  objects/s. Further analysis of data from the first 5 s of the acquisition showed the maximal rate of 897.20  objects/s. After applying morphological filtering conditions, 107,631 objects were identified as RBCs and kept for analysis. Each dataset of 10,000 frames required an average of 1.55 s for calibration and 33.33 s for acquisition.

**Fig. 5:**
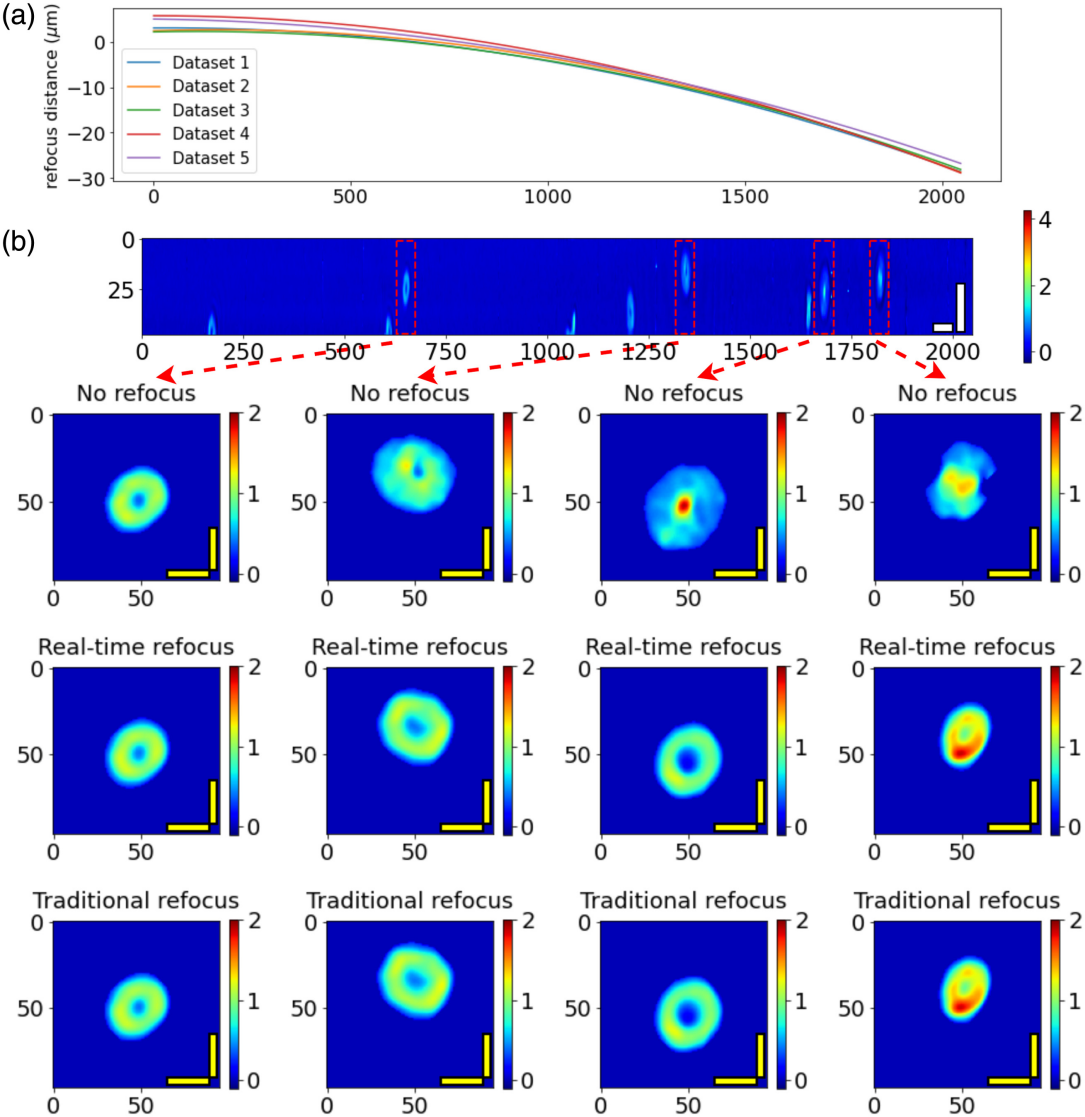
(a) Refocus curve of all five datasets; (b) phase map of a processed frame, together with 4 RBCs identified and refocused [white scale bars: 20  μm in x-axis, 10  μm in y-axis; yellow scale bars: 5  μm; colorbars: phase (rad)].

The refocusing of the QPM cell images was conducted using the calibration curve described above. Figure 5(a) shows the refocusing curves calibrated from five different datasets. The closeness of these curves demonstrates that there was no significant movement of the refocus plane across several minutes. The general trend is that the calibration curve was tilted significantly to the lower right, resulting in the right side of the FOV being out of focus, as shown by the larger refocusing distance. Figure 5(b) shows a single processed frame from the collected dataset, from which four RBCs were identified. By comparing the cell images before and after refocusing, we found that the cells on the left side of the FOV were generally in focus with clear RBC structure without refocusing. The cells on the right side of the FOV were blurred to varying degrees due to defocus. However, the RBC images of these cells could be recovered by refocusing to the calibrated distance.

### 3.2 Statistical Analysis of RBCs

**Fig. 6:**
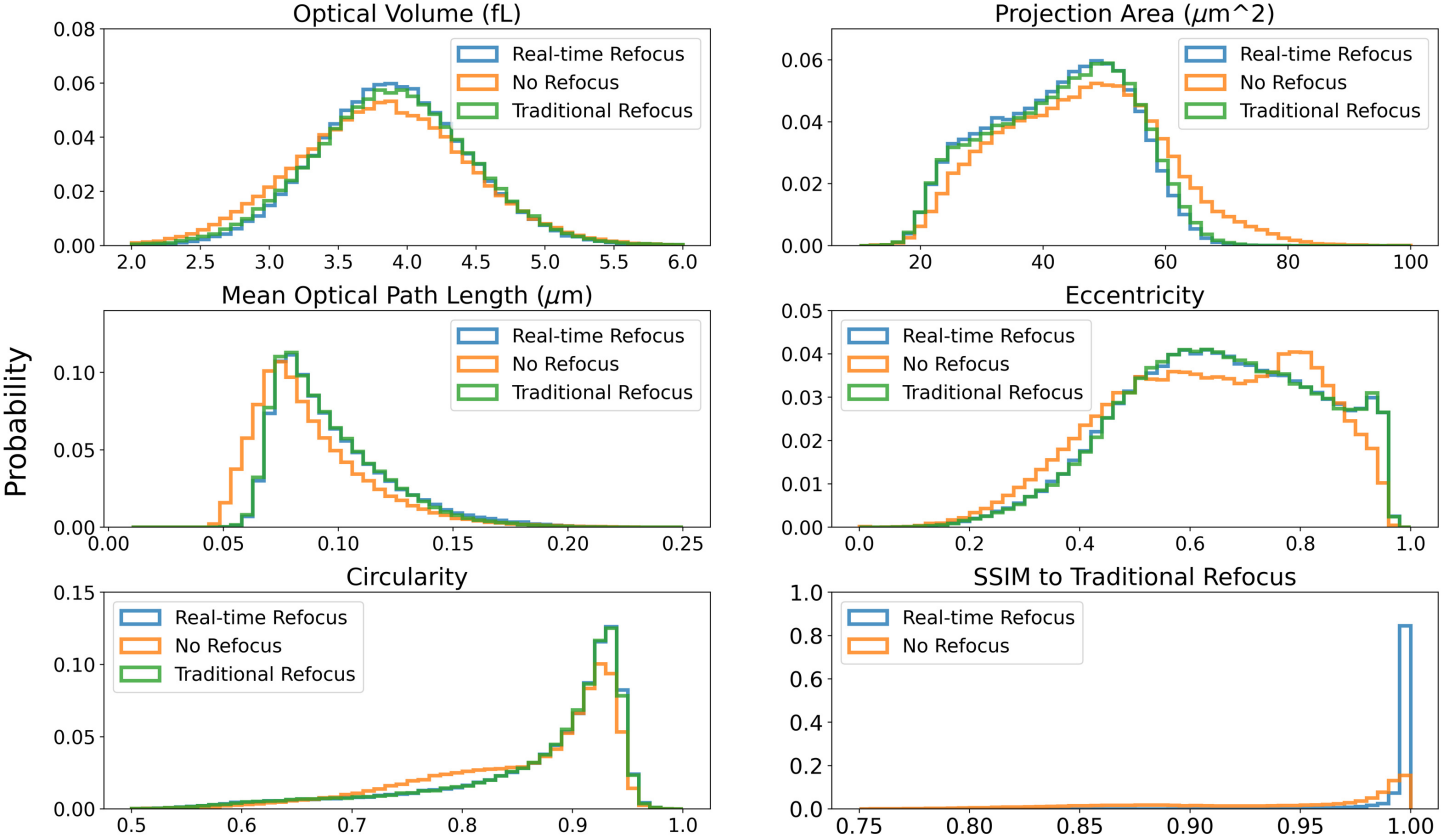
Histograms of morphological parameters and SSIM from different refocusing methods.

To characterize the fidelity of the image processing, the error rate of every captured cell’s morphological parameter and the RMSE and SSIM were calculated by comparing each RBC image before and after refocusing with the traditional refocusing result. Figure 6 shows the histograms of different refocusing results. The real-time refocusing method provides almost the same histogram compared with the traditional method. For several of the parameters, it is difficult to see a difference between the two histograms due to their overlap. The distributions of OV and projection area also match our previous analysis of normal RBCs.^5^ Table 2 shows the results of the observed mean error and the 95th percentile. For most of the metrics, the real-time refocusing can provide a reduction in mean error by a factor larger than 3, and for more than 95% of the refocused cells, an error rate less than 10% is achieved. The average SSIM of the refocused cells is up to 0.996, which proves our real-time refocusing restores nearly all RBC details.

**Table 2:** Comparison of morphological parameters before and after real-time refocusing to the traditional refocusing, with the improvement factor given.

Refocus	Mean			95th percentile		
	Before	After	Factor	Before	After	Factor
OV (% error)	7.96%	2.56%	**3.11**	24.3%	9.29%	**2.62**
Area (% error)	9.62%	1.71%	**5.63**	34.0%	4.32%	**7.87**
Mean OPL (% error)	10.9%	1.85%	**5.89**	30.1%	6.09%	**4.94**
Eccentricity (absolute diff.)	0.0491	0.00888	**5.52**	0.181	0.0296	**6.11**
Circularity (absolute diff.)	0.0345	0.00870	**3.97**	0.149	0.0235	**6.34**
RMSE of OPL (μm)	0.0111	0.00219	**5.07**	0.0299	0.00775	**3.86**
SSIM	0.938	0.996	**NA**	0.818	0.983	**NA**

Note: bold entries emphasize the improvement factor before and after the refocusing.

### 3.3 Speed Test Results

Table 3 shows the speed test results of the average processing time of each frame, including the time required for the different processing steps for four different concentrations of RBCs, given as the number of cells per second. The time required for cell refocusing and cell analysis was calculated by multiplying the average execution time of a single cell by the average number of cells in one frame. Notice that these steps are conducted in parallel; thus, the overall processing time is limited by the slowest step instead of the summation time of all steps.

**Table 3:** Averaged processing time per frame with different RBC concentrations.

	No cells (ms)	441 cells/s (ms)	909 cells/s (ms)	1284 cells/s (ms)
Phase retrieval and residues	0.97	0.96	0.95	0.95
Branch cuts	0.72	0.73	0.73	0.73
Unwrapping	0.81	0.80	0.80	0.77
Post-processing	0.34	0.31	0.30	0.31
Cell identification	NA	0.11	0.11	0.11
Cell refocusing	NA	1.84	2.58	3.25
Cell analysis	NA	0.65	0.97	1.20
**Whole pipeline**	**0.98**	**1.86**	**2.66**	**3.33**

Note: bold entries emphasize the whole pipeline execution time per frame at different cell concentrations.

As the cell concentration increased, the QPM processing time was almost constant, whereas the time spent on cell refocusing and analysis increased and affected the overall processing time. As the acquisition rate of the camera is 300 fps, which corresponds to 3.33  ms/frame, the speed test results show that the maximum cell processing speed is ∼1200  cells/s.

## 4 Discussion

We have shown that the implementation of an HC system with real-time acquisition and processing on an embedded computer provided 4096×96  pixel interferograms, 2048×48  pixel phase videos, 96×96  pixel segmented and refocused cell images and extracted four morphological parameters for each cell for primary filtering. When the cell extraction function is disabled and only the QPM processing is executed, the average processing time of a 2048×48  pixel frame was 0.98 ms. In Table 4, we listed the QPM processing performance of our system and previous GPU-employed studies.

**Table 4:** Comparison of previous GPU-employed QPM processing studies with the reported system.

Author (year)	GPU and FP32 (TFLOPS)	Output size (pixel)	Processing time (ms)	Speed (pixels/ms)
Pham et al.^9^	GTX 470M (0.616)	512 × 512	2.60	100,825
Morales et al.^17^	RTX 3060 (12.738)	1280 × 960	11.11	108,864
Bai et al.^18^	Jetson Nano (0.236)	512 × 512	540	485
Li et al.^19^	GTX1650 (2.984)	512 × 512	3152	83
Park et al.^20^	Not Specified	256 × 256	502.1	131
**Reported system**	**Jetson Orin Nano (2.089)**	**2048 × 48**	**0.98**	**100,310**

Note: bold entries emphasize the features of our reported system.

In comparison to our result, Pham’s first implementation of Goldstein’s algorithm on a GPU^9^ achieved an average processing speed of 100,825  pixels/ms, which is still the state-of-the-art processing time for QPM processing using this algorithm. This study utilized a different GPU that was less powerful than the Jetson Orin Nano. However, our input interferogram is twice the size of the output phase video, and our pipeline has additional post-processing steps, which increase the required processing time. Therefore, it is not possible to directly compare the performance of the two algorithms. Nevertheless, this still shows that the presented QPM system has a comparable processing speed to the former state of the art.

Recently, Morales et al.^17^ reported a GPU-accelerated QPM processing software called HoloStream, which can process 1280×960  pixels QPM data at 90 fps from pre-recorded videos. When capturing and displaying in real time, its frame rate was reduced to 11 fps. This software was tested on an NVIDIA RTX 3060 desktop GPU. In comparison, the HC system can achieve comparable performance on an embedded NVIDIA Jetson Orin Nano Super platform, on which the GPU has lower performance but a significantly smaller size.

As a system-on-module (SoM), the Jetson Orin Nano can be integrated into a portable device, and its latest price is only $249. As this study has proven Jetson’s ability to control the HC system, capture QPM data, and process data in real-time, it is possible to build a low-cost and portable system using this platform. Another advantage of the Jetson platform is that its GPU and CPU share the same unified memory, which allows data transfer with extremely low latency, whereas desktop GPUs require additional data transfer latency and cannot flexibly allocate CPU and GPU tasks during processing to maximize resource utilization. Previous research by Bai et al. on QPM processing on an embedded system used an even lower-end Jetson Nano module.^18^ However, their reported processing time of a 512×512  pixel frame was up to 540 ms, which was not suitable for a high-throughput HC system.

Deep learning is another potential method for QPM image processing and may better handle phase jumps by identifying object features. Although previous studies have proven the precision of deep-learning-based phase unwrapping, execution time still limited their feasibility on high-throughput QPM systems. For example, Li et al. reported an execution time of 3.152 s on a 512×512 phase map.^19^ Furthermore, Park et al. reported that it took 0.5 s to process a 256×256  pixel phase map.^20^ Therefore, lightweight models are required to speed up QPM processing deployed on real-time, high-throughput systems.

The current QPM system offers practical advantages for imaging human RBCs for clinical applications. For example, in 2013, Pham et al.^21^ reported a QPM system that can perform 1024×1024  pixel image reconstructions of RBCs at 40 fps and extracted morphological parameters in real time. However, this system could only observe a small number of cells by manually adjusting the focus and moving the FOV. In 2021, Nissim et al.^22^ conducted real-time blood cell classification with a holographic microscopy system and analysis using machine learning, but the throughput was only 15  cells/s, limiting the size of the cell population that could be effectively studied. In comparison, the HC system presented here can automatically acquire large amounts of data at a video rate of 300 fps and a cell rate of 1200  cells/s. In addition, the processing approach developed here performs digital refocusing and extracts morphological parameters in real time. This allows us to compensate for deviations from manual focusing or platform tilt and obtain precise morphological parameters. Although this information has not been used to develop a real-time machine learning classification algorithm at this time, the morphological parameters extracted can be used to conduct a quick statistical analysis of the cell population. By extracting more parameters and deploying machine learning,^7^ the HC system should be able to classify hundreds of RBCs per second.

## 5 Conclusion

In this study, we demonstrate an SoM approach for real-time QPM data processing. A CUDA phase processing algorithm and parallel computing are leveraged to achieve significantly accelerated phase processing. Jetson Orin Nano provides a relatively low-cost embedded platform to perform accelerated parallelized processing. We implement a real-time data processing pipeline on a high-throughput HC system, which automatically collects refocused phase images and calculates basic morphological parameters. The real-time HC system offers the ability to rapidly image and analyze a large number of RBCs. This processing acceleration potentially improves the HC system’s diagnostic capability to detect conditions such as sickle cell disease. The results from the healthy RBC sample presented here show that the real-time processing pipeline achieves high structural similarity with low deviation relative to a traditional processing method. Compared with traditional blood screening methods, the HC system provides several additional morphological parameters of RBCs, which are highly suited for machine learning applications. In clinical settings, machine learning algorithms could be incorporated into the processing pipeline to automatically classify collected RBC images based on the measured morphological parameters. As the system control and processing pipeline are realized on a Jetson embedded platform, we expect this study to benefit the development of a portable, low-cost HC system.

## 6 Appendix A: Processed Holographic Cytometry Video

Video 1 shows the captured and processed phase video in 1 s (300 frames). Side artifacts are observed on both sides of the cell, which is due to the presence of the cell slightly raising the curve when fitting the background in Sec. 2.2.3. As the video is only used to identify the cell and background coordinates, and the output phase information comes from the reconstructed complex optical field, these artifacts will not affect the accuracy of the output cell images.

**Video 1** Processed holographic cytometry video in 10 fps (Video 1, MP4, 1.13 MB [URL: https://doi.org/10.1117/1.BIOS.3.1.012902.s1]).

## 7 Appendix B: Bead Calibration Details

Polystyrene bead calibration was conducted to demonstrate the accuracy of our system’s phase measurement. Details of the experimental method and results can be found in the Supplementary Material.^23^ A processed video of flowing beads is shown in Video 2, where 3,064 bead images were obtained from the reported system. Notice that the video was not refocused, so some beads are blurred.

**Video 2** Processed holographic cytometry video of beads in 30 fps (Video 2, MP4, 446 KB [URL: https://doi.org/10.1117/1.BIOS.3.1.012902.s2]).

## Supplementary Material

10.1117/1.BIOS.3.1.012902.s01

10.1117/1.BIOS.3.1.012902.s1

10.1117/1.BIOS.3.1.012902.s2

## Data Availability

Data and code used to generate figures and tables are available. One of the RBC datasets and the processing software are also available. The repository of the code and data availability section can be found here: https://doi.org/10.7924/r49s21900.
