# Supplementary material for: Real-time processing of high-throughput quantitative phase microscopy data using a Jetson Orin Nano
Source: Biophotonics Discov. 2025 Oct 21;3(1):012902. doi: 10.1117/1.BIOS.3.1.012902 (PMC13052479; doi:10.1117/1.BIOS.3.1.012902)
Supplement: Supplementary file 1 [file BIOS_003_012902_SD001.pdf]

Supplementary Material

S1 System Calibration on Polystyrene Beads

S1.1 Beads Calibration Method

To evaluate the accuracy of our phase measurements, we imaged flowing 5.1 ±0.5 µm polystyrene beads (n = 1.585 at 640 nm, Thermo Scientific, MA) in index-matching liquid (n = 1.546 at 640 nm, Cargille, NJ). The manufacturer gives an expanded uncertainty for the bead's mean diameter as ±0.3 µm (k=2).

The diameter of each bead was estimated by measuring the volume given by the formula:

$$V = \frac{OV}{\Delta n} \tag{S1}$$

where OV is the optical volume calculated from Eq. (5) and Eq. (6), and Δn is the refractive index difference of the beads and liquid. The diameter can be calculated by the sphere volume formula:

$$d = 2 * \sqrt[3]{\frac{3V}{4\pi}} \tag{S2}$$

The captured bead images were also compared with a simulated sphere with matched diameter. The previous study of Mohammad et. al. [23] proved that a phase shift will occur due to the finite lateral resolution of a real optical system and it effectively acts as a low-pass filter in the reconstruction. To simulate this blurring effect, we applied the same Butterworth low-pass filter as was used in the phase retrieval step. The phase profile of the captured bead was then compared with both the ideal simulated sphere and the filtered simulated sphere.

S1.2 Beads Calibration Result

A processed video of flowing beads is shown in Video 2, where 3,064 bead images were obtained from the reported system. Notice that the video was not refocused, so some beads are blurred.

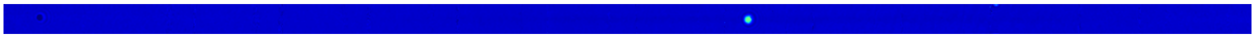

Video 2 Processed holographic cytometry video of beads in 30 fps (MPEG, 0.45 MB).

The histograms of the measured bead diameters are shown in Fig. S1. The mean and standard deviation of the measured diameters on the real-time processing system is 4.878±0.384 µm. When

processed with traditional methods, the diameter obtained is 4.870±0.385 µm. Both results are within the manufacturer's declared diameter uncertainty range with an error of less than 5%.

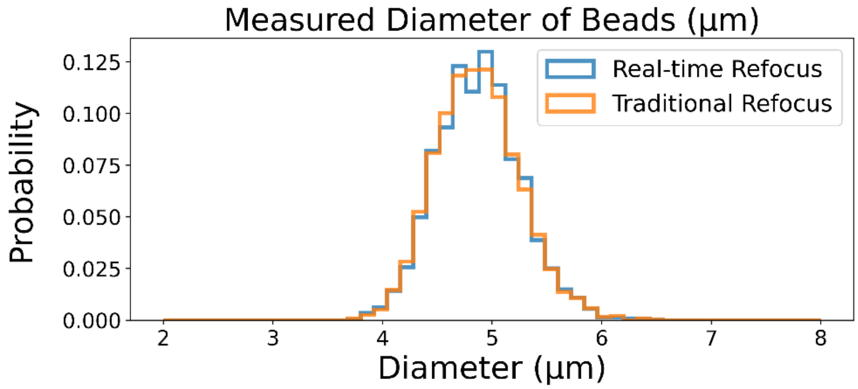

Fig. S1 Histograms of measured diameters of 5.1µm beads from different refocusing methods

Fig. S2(a) shows a bead phase map and Fig. S2(b) provides the phase profile along the diameter compared with the ideal simulated sphere and the filtered simulated sphere, where the bead's phase curve matches with the low-passed filter sphere and the same blurring effect can be observed at both curve's edges.

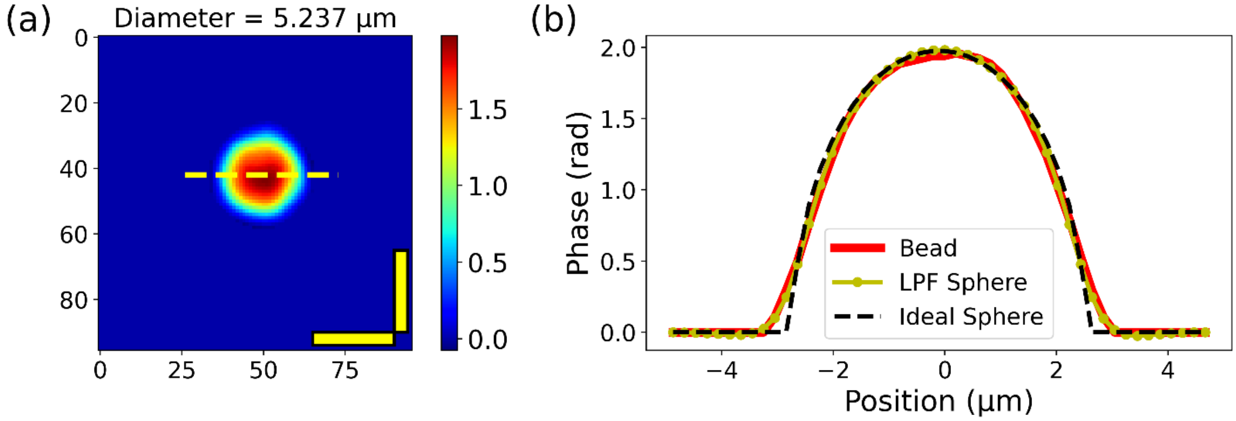

Fig. S2 (a) A captured bead phase map; (b) phase curves of imaged bead and simulated spheres along the diameter. (Scale bars: 5 µm; color bar: Phase (rad))

The results of bead diameter measurement and phase curve matching demonstrated our system's capability to measure phase delays in flowing samples accurately.
